# Investigations of Diclofenac Sorption on Intact and Modified *Chlorella vulgaris* Biomass with pH-Switchable Desorption

**DOI:** 10.3390/ijms27031413

**Published:** 2026-01-30

**Authors:** Ivan Liakh, Adrian Szewczyk, Magdalena Prokopowicz, Magdalena Narajczyk, Anna Aksmann, Darya Harshkova, Bartosz Wielgomas

**Affiliations:** 1 Department of Toxicology, Faculty of Pharmacy, Medical University of Gdańsk, Al. Gen. Hallera 107, 80-416 Gdansk, Poland; ivan.liakh@gumed.edu.pl; 2 Department of Physical Chemistry, Faculty of Pharmacy, Medical University of Gdańsk, Al. Gen. Hallera 107, 80-416 Gdansk, Poland; adrian.szewczyk@gumed.edu.pl (A.S.); 3 Bioimaging Laboratory, Faculty of Biology, University of Gdańsk, Ul. Wita Stwosza 59, 80-308 Gdansk, Poland; magdalena.narajczyk@ug.edu.pl; 4 Department of Plant Experimental Biology and Biotechnology, Faculty of Biology, University of Gdańsk, Ul. Wita Stwosza 59, 80-308 Gdansk, Poland; anna.aksmann@ug.edu.pl (A.A.); darya.harshkova@ug.edu.pl (D.H.)

**Keywords:** *Chlorella vulgaris*, diclofenac sorption, adsorption isotherms, lipid-extracted biomass

## Abstract

The growing interest in sustainable and structurally diverse sorbent materials has intensified the search for effective biosorbents that can complement or replace conventional adsorbents. This work presents the potential use of *Chlorella vulgaris* dried biomass and its modifications (ultrasound-treated, lipid-extracted, and combined forms) for diclofenac (DCF) sorption from aqueous solutions. It was demonstrated that sorption efficiency significantly depends on the solution’s pH. Lowering the pH from 6 to 2 increases the sorption from 5% to 68%, while 99% desorption occurred at pH 9. The adsorption isotherms for intact biomass and after lipid extraction (CV-E2) are best described by the Langmuir and Freundlich models; for ultrasonically treated biomass (CV-E1) by the Temkin model; and for ultrasound-assisted solvent extraction (CV-E3) by the Dubinin–Radushkevich model. These findings demonstrate that cellular lipids and particle characteristics critically govern sorption mechanisms, highlighting dried *Chlorella* biomass as a structurally and chemically tunable biosorbent. Importantly, the key sorption experiments were performed under strongly acidic conditions (pH 2), which differ from typical wastewater or surface water matrices. Therefore, the presented results should be regarded as a proof of concept illustrating the mechanistic potential of dried Chlorella biomass as a tunable sorptive material, with prospective relevance for separation science and laboratory-scale analytical applications rather than direct environmental remediation.

## 1. Introduction

Non-steroidal anti-inflammatory drugs (NSAIDs) are among the most common drugs found in the environment due to their widespread use, availability, and partial resistance to wastewater treatment technologies [1]. Their presence in the aquatic environment, even at trace levels, has been associated with toxic effects on non-target organisms, including inhibition of algal growth, impairment of fish reproduction, and bioaccumulation in aquatic food chains and impact on drinking water quality, highlighting the need to address their removal from natural matrices [2].

Among NSAIDs, diclofenac (2-[2-(2,6-dichloroanilino)phenyl]acetic acid; DCF) is one of the most extensively studied pharmaceutical compounds due to its prevalent use in human and veterinary medicine, high environmental stability, and potential ecotoxicity. DCF exerts adverse effects on both aquatic and terrestrial organisms. In aquatic environments, DCF toxicity has been documented in bacteria, algae, invertebrates, and fish, manifesting as tissue damage, impairment of physiological functions, and changes in community structure at environmentally relevant concentrations (ng/L–µg/L) [3]. A well-known example of negative impact on non-target terrestrial species is the collapse of vulture populations in Pakistan, which resulted in population declines exceeding 98% in several Gyps species [3]. Recent studies further demonstrate that DCF affects multiple trophic levels by inhibiting microalgae (*Pseudokirchneriella subcapitata*) growth, disrupting zooplankton (*Daphnia curvirostris*) reproduction, and inducing lethal and sublethal effects in fish (*Danio rerio*) embryos and larvae [4].

DCF is frequently used as a model compound in studies addressing sorption, partitioning, and interaction mechanisms between pharmaceuticals and solid materials. Of the materials tested for DCF sorption, activated carbon (often derived from plant sources) remains the most widely used and effective option [5,6,7,8]. Recently, however, the use of intact and modified microalgal biomass has attracted growing attention as a lower-cost, bio-based alternative to activated carbon [9,10]. Microalgal biomass can be applied for the removal of organic contaminants either in the form of living cells [11] or as inactivated (non-living) material [12], and these two approaches differ fundamentally in their underlying mechanisms. In living algal systems, contaminant removal may involve a combination of surface adsorption, active uptake, intracellular accumulation, metabolic transformation, and, in some cases, biodegradation [11]. As a result, the observed removal efficiency reflects the interplay of biological activity and physicochemical interactions, which complicates mechanistic interpretation and limits control over process reproducibility [11,13].

In contrast, non-living algal biomass acts exclusively as a passive sorbent, in which contaminant uptake is governed by physicochemical processes such as surface adsorption, hydrophobic partitioning, and pore-related interactions. It should be emphasized that the present study exclusively investigated dried, non-living *Chlorella vulgaris* biomass and its modified forms.

Algae are inexpensive adsorbents rich in functional groups such as amino, carboxyl, sulfate, and hydroxyl, making them effective in adsorption processes [14,15]. Algae biomass contains proteins, polysaccharides, and lipids, making it suitable for the removal of contaminants including heavy metals, antibiotics, dyes, aromatic hydrocarbons, and herbicides [16,17,18,19]. Live algae are also being explored for the removal of potentially toxic elements (mainly heavy metals) and pharmaceutical and personal care products from wastewater [10,19,20,21]. In particular, unicellular algae such as *Chlorella* sp., *Nannochloropsis* sp., *Scenedesmus* sp., *Parachlorella* sp., *Picocystis* sp., and *Graesiella* sp. have demonstrated high DCF removal efficiency (ranging from 52–91% removal) [22,23,24,25,26].

Among the above-mentioned microalgae, *Chlorella vulgaris* is one of the most studied microalgae species, known for its rapid growth, high biomass yield, and ease of cultivation. Dry *Chlorella* biomass has long been produced on an industrial scale: it is added to dietary supplements, cosmetics, and animal feed, and is used for biofuel production, after which a significant amount of residual biomass remains [27,28,29,30]. Another advantage is that *Chlorella* can be grown on nutrients recovered from wastewater, making the entire process significantly more environmentally friendly [31].

The majority of studies on biosorbent materials have focused on their application in the removal of contaminants from aqueous systems, particularly wastewater and environmental matrices. While this remediation-oriented perspective has dominated the literature, it represents only one aspect of the potential utility of biosorbents. Due to their chemical heterogeneity, abundance of functional groups, and tunable surface properties, biosorbent materials also offer promising opportunities in non-remediation contexts, including separation science and analytical chemistry.

To the best of our knowledge, this is one of the first studies to systematically investigate the mechanism of DCF sorption by dried *Chlorella* biomass, including the influence of its lipid content, particle characteristics, and surface properties. Additionally, a series of experiments were performed to explore the mechanism behind that process, for which we selected optimal conditions for using dry biomass and compared adsorption isotherms for sorbents with different lipid content and different particle characterization. In addition, to maximize economic benefits, we investigated the possibility of reusing sorbents and compared the efficiency of biomass sorption after lipid extraction, which allows the use of large volumes of biomass waste generated during biofuel production [29]. Moreover, the findings of this work may support future studies on the applicability of *Chlorella* biomass in laboratory-scale processes, including analytical chemistry, as an environmentally friendly alternative to sorbents used in dispersive or conventional solid-phase extraction.

## 2. Results and Discussion

### 2.1. Physicochemical Characterization of Sorbents

#### 2.1.1. Microscopy

**Figure 1:**
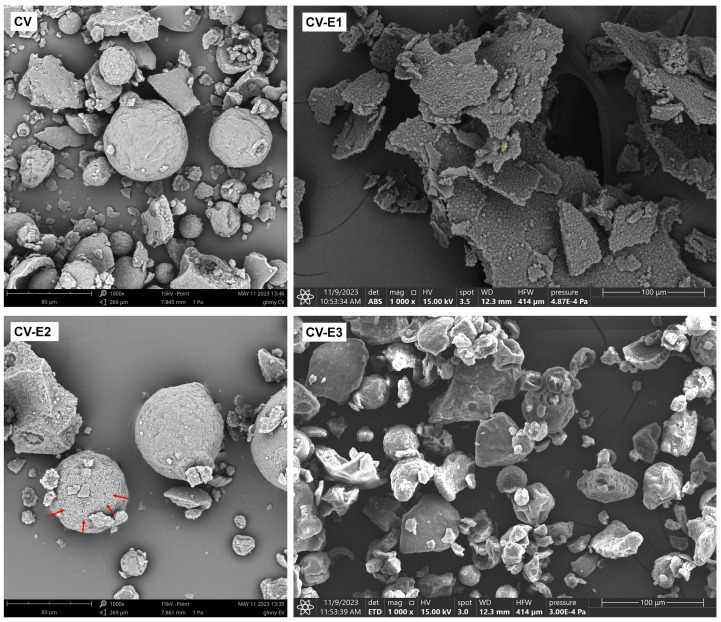
SEM images of dry *Chlorella* powder (CV), *Chlorella* powder after sonication (CV-E1), *Chlorella* powder after Soxhlet extraction (CV-E2), and *Chlorella* powder after ultrasound-assisted solvent extraction (CV-E3), magnification 1000×. Red arrows indicate pores formed on the surface of cellular aggregates following Soxhlet extraction. The yellow “+” symbol visible in the center of the CV-E1 image is a software-generated marker and has no physical or analytical significance.

Electron microscopy revealed that although *Chlorella cells* are typically spherical and range from 3 to 10 µm in diameter, in the intact *Chlorella* powder (CV), most of them were assembled into irregular clusters, among which spherical aggregates (20–80 µm) were also observed (Figure 1 and Figures S6 and S7). Such aggregates have been reported previously and likely formed due to the cell adhesion during harvesting and drying [32,33,34]. After sonication (CV-E1), the previously described clusters and large aggregates disappeared, and the cells formed thin flat structures, possibly as a result of aggregation of cells and intracellular debris during the drying process (Figure 1 and Figure S7). Individual cells had more irregular shape (not round) compared to untreated CV, and the space between cells was filled with debris (Figure S5). Soxhlet extraction (CV-E2) did not cause the disintegration of cellular aggregates and clusters, while multiple perforations on the aggregates’ surface were observed (Figure 1 and Figure S7). In the CV-E3 group, after ultrasound-assisted solvent extraction, similar changes were observed as in CV-E2 group, except that larger surface aggregates were fragmented into smaller clusters due to ultrasonic exposure.

#### 2.1.2. Physical and Molecular Characteristics of Sorbents and the Efficiency of Extraction of Lipids and Pigments

Flow cytometry analysis of sorbents in brightfield mode revealed a decrease in particle number with the area feature (threshold mask on cell contour, used to quantify and compare cell size) of 40–200 μm^2^ in the CV-E2 groups compared to CV. This decrease can be explained by the larger number of particles outside the range of comparison (with an Area less than 5 µm^2^).

**Figure 2:**
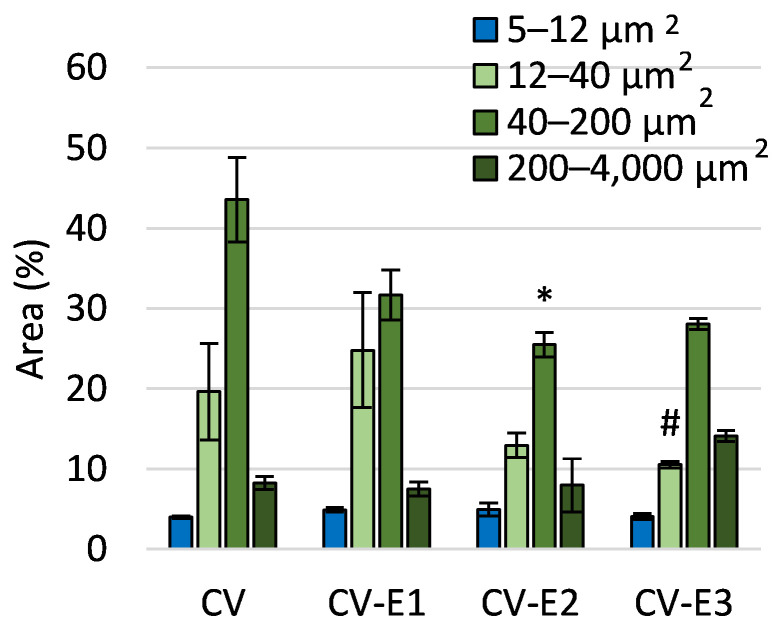
The comparison of area features as a morphological characteristic of the studied sorbents obtained using flow cytometry. Values are the mean ± SEM; *, #—significant difference compared with CV and CV-E1 groups, respectively, at *p* < 0.05 (Kruskal–Wallis, post hoc Dunn’s test).

The decrease in the number of particles with an area of 12–40 μm^2^ in the CV-E3 group compared to CV-E1 can be attributed to the lower cell disruption efficiency in organic solvents compared to water (Figure 2). This may result from the suppression of cavitation effects due to higher vapor pressure, lower surface tension, and increased viscosity, which reduces the overall efficiency of ultrasonic treatment [35]. Additionally, the formation of free radicals during cavitation is much more pronounced in aqueous systems, while organic solvents tend to quench or limit radical generation, further lowering the disruption efficiency [36]. It is worth noting that ultrasound treatment (CV-E1) resulted in a greater number of morphological alterations compared to other groups (Table S6).

Chlorophyll autofluorescence at Ex = 488 nm (detected band 640–745 nm) showed significant decrease in relative fluorescence intensity (1000–50,000 a.u.) after solvent extraction (CV-E2), mainly due to the removal of lipophilic pigments (Figure S8). A similar trend of relative fluorescence intensity reduction (50–50,000 a.u.) observed in the CV-E1 and CV-E3 groups also may be attributed to chlorophyll loss during extraction [37,38].

**Figure 3:**
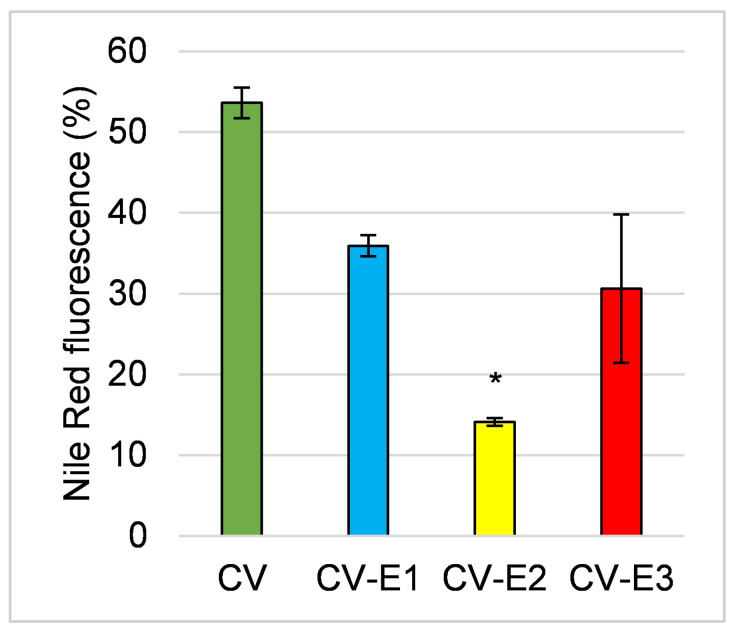
The comparison of fluorescence activity of the studied sorbents after staining with Nile red. Values are the mean ± SEM; *—significant difference compared with intact *Chlorella* powder at *p* < 0.05 (Kruskal–Wallis, post hoc Dunn’s test).

Nile red staining (detected band 560–595 nm) also revealed changes in lipid content and cell integrity [39,40], with a notable reduction in fluorescence intensity across all groups, particularly after Soxhlet extraction (CV-E2), indicating lower lipid content (Figure 3).

#### 2.1.3. Porosity

The specific surface area (S_BET_), mean pore diameter (D_BJH_), and pore volume (Vp) of the studied sorbents are presented in Table 1. Based on the obtained nitrogen adsorption–desorption results, the intact powder of *Chlorella vulgaris* (CV) was characterized as a nonporous material. Contrary to intact material, relatively more porous sorbents were obtained after the modification, despite the type of used procedure. The modified sorbents (CV-E1, CV-E2, CV-E3) were characterized by the mesoporous structure, as the mean D_BJH_ values were in the range of 2–50 nm [41]. The use of the extraction by sonication with organic solvents (CV-E2) resulted in the sorbent with the relatively highest S_BET_ and Vp values, among all investigated materials.

#### 2.1.4. ATR-FTIR

**Figure 4:**
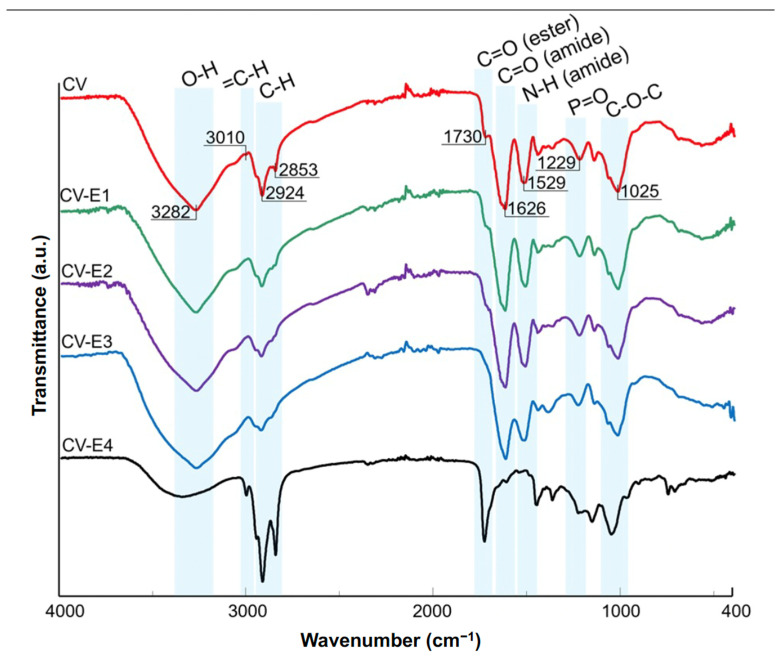
ATR-FTIR spectra of *Chlorella vulgaris* samples before (CV, red) and after various modification methods (CV-E1, green; CV-E2, purple; CV-E3, blue), together with lipid fraction sample (CV-E4, black).

The ATR-FTIR results of CV, CV-E1, CV-E2, and CV-E3 samples, together with the spectrum of sample extract (CV-E4), are presented in Figure 4. The spectrum of intact *Chlorella vulgaris* sample (CV) revealed the dominant bands at 3282, 2924, 1626, 1529, 1229, and 1025 cm^−1^ characteristic for O-H stretching, aliphatic C-H stretching, C=O stretching (amide I of proteins), N-H bending (amide II of proteins), P=O stretching (phosphodiesters of nucleic acids), and C-O-C stretching modes of polysaccharides, respectively [42,43]. The band at 1025 cm^−1^, present in the CV-E4 sample extract, can be explained by polysaccharide residual particles in the sorbent that remained after extraction. In the case of lipid extraction, two additional bands at 3010 (=C-H stretching) [44] and 1730 cm^−1^ (C=O stretching of esters) [42] were of particular interest. The intensities of these bands decreased significantly in the spectra of samples after extraction (Figure 4, CV-E1, CV-E2, and CV-E3), whereas their strong presence in the spectrum of sample extract (CV-E4) was reported. These observations proved the successful extraction of lipids from intact *Chlorella vulgaris* material. These findings were in agreement with other studies that focused on the determination of lipid content obtained from algae [42,44]. Interestingly, similar results were reported not only for algae samples but also for fungal biomasses after lipid extraction [45].

### 2.2. Sorption Studies

Several species of microalgae have been investigated as biosorbents for the removal of pharmaceuticals, including NSAIDs, due to their high surface reactivity and affinity for organic pollutants. Thus, *Scenedesmus obliquus* biomass showed high efficiency in removing pharmaceutical pollutants [46], including NSAIDs [47]. In another study on the removal of DCF from water, two different microalgae strains, namely, *Synechocystis* sp. and *Scenedesmus* sp., reached the Langmuir maximum capacity (*q_L_*_(*max*)_) values of 20 and 28 mg/g, respectively [48]. In both cases, the equilibrium results are best fitted by the Langmuir isotherm, which indicates a difference in sorption mechanisms (monolayer adsorption) compared to activated carbon. At the same time, *Chlorella* biomass used as a biosorbent for phenolic compounds [49] also showed high efficiency in removing metallic impurities from wastewater [50,51]. Based on the fact that live cultures of *Chlorella* have shown a high degree of removal of NSAIDs and DCF [24,52,53,54], and are also suitable for short-term adsorption of DCF (fitting to the Freundlich model) [22], there is a logical interest in the efficiency of DCF removal using dry *Chlorella* biomass, since biosorption usually has a higher removal rate compared to bioaccumulation [55].

At the same time, other experiments have shown that the efficiency of drug removal from an aqueous solution strongly depends on the lipid content of the sorbent, as well as on the physicochemical conditions of the environment. Al-Mashhadani et al. showed that using extracted *Chlorella* biomass (double Soxhlet extraction with ethanol and chloroform), 89.9% adsorption of ciprofloxacin could be achieved; in addition, after extraction, the surface area of the biomass increased, and important functional amide and carboxyl groups were retained [56]. Suárez-Martínez et al. showed that the adsorption capacity of tetracycline in dry *Chlorella* sp. biomass after the lipid extraction using a mixture of chloroform/methanol (2:1) and ultrasound (1 h), followed by Soxhlet extraction with hexane, was higher than that of the untreated biomass [57], wherein Angulo et al. showed a reduction in cephalexin uptake by extracted *Chlorella* biomass [58]. Therefore, to assess how particle size and lipid content affect sorption, we prepared modified sorbents that enabled us to examine the individual and combined effects of these two factors (Table S2).

#### 2.2.1. Preliminary Studies

Comparison of sorption at three different pH values (2.0, 4.0, and 6.0) confirmed the inverse dependence of sorption and pH; in the case of pH 2.0, the reduction in the DCF level reached 30% (for CV-E4) compared to the control. At the same time, the concentration of DCF at pH 6.0 decreased only in the lipid fraction (CV-E4) (Figure S1). Since the neutral form of DCF is more hydrophobic, its lipophilicity and affinity for the lipid fraction of the algal biomass are higher at pH 2 than at pH 6. This explains the enhanced sorption observed at lower pH values, as the neutral form can interact more effectively with hydrophobic sites within the biomass, and this also explains the slightly better sorption (at pH 2.0) in materials with a higher lipid content (CV and CV-E1) [59].

When using different sorbent concentrations (1, 2.5, 5, 10, and 20 g/L), it was shown that at a sorbent dose above 10 g/L, complete sorption of DCF (1 mg/L) from the solution is observed (Figure S2), suggesting potential for practical application in wastewater treatment. Reported DCF concentrations in municipal and hospital effluents typically range from a few ng/L to several μg/L, depending on the source and region [60]. Moreover, the pH of such wastewater is usually close to neutral or slightly alkaline (pH 6.2–9.8) [61], whereas the highest sorption efficiency of *Chlorella* biomass is observed at acidic pH. Thus, exploring strategies for lowering the pH of natural or wastewater systems (for example, through the use of environmentally safe buffering agents) appears promising and warrants further investigation. Nevertheless, a working sorbent concentration of 0.5 g/L was chosen to determine adsorption isotherms under controlled conditions, since it does not provide complete sorption.

A study of the sorption and desorption dynamics of DCF on CV powder showed that at both concentrations of CV (0.5 and 5 g/L), the maximum sorption occurred in the first 5 min (Figure S3).

#### 2.2.2. Adsorption Isotherms

**Figure 5:**
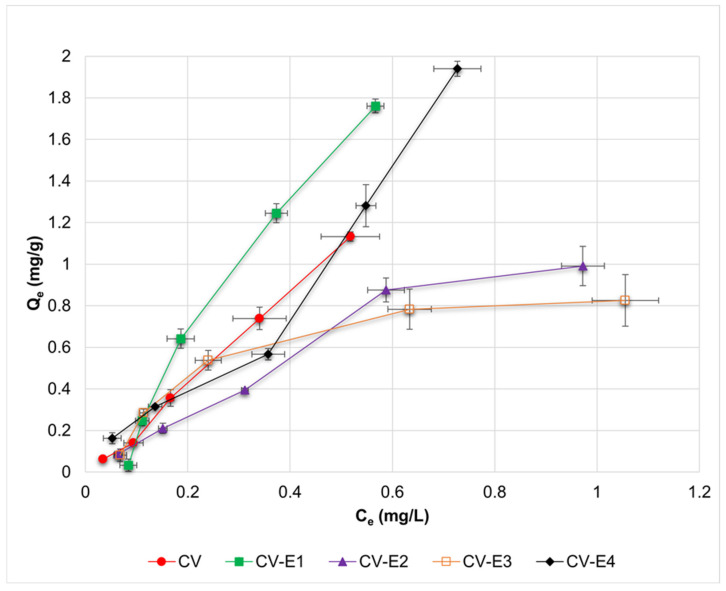
Adsorption isotherms of DCF for investigated sorbents (CV, CV-E1, CV-E2, and CV-E3), together with data obtained for sample extract (CV-E4). Horizontal and vertical error bars denote standard deviations of *C_e_* and *Q_e_*, respectively.

The adsorption isotherms of DCF and the calculated parameters from Langmuir, Freundlich, Dubinin–Radushkevich, and Temkin adsorption models for investigated sorbents are presented in Figure 5 and Table 2, respectively. Based on the obtained *R*^2^ values (Table 2), it might be observed that experimental results reported for CV and CV-E2 were best fitted by the Langmuir and Freundlich models; for CV-E1 by the Temkin model; and for CV-E3 by the Dubinin–Radushkevich model, accordingly. In the case of the Langmuir model, the negative, and thus uninterpretable, values of *q_L_*_(*max*)_ and *K_L_* were obtained for CV, CV-E1, and CV-E3 sorbents. Such values might result from two factors: low concentrations of DCF used during the sorption studies, which were limited by the low solubility of DCF in an acidic medium (1.70 ± 0.12 mg/L) and due to the relatively low porosity of these sorbents (Table 1). Nonetheless, positive values of *q_L_*_(*max*)_ and *K_L_* parameters were reported for sorbent with extracted lipid fraction (CV-E2), most likely due to its highest porosity (Table 1).

Surprisingly, it was also possible to estimate the values of Langmuir parameters for sample extract (CV-E4), most probably due to the presence of residual particles of CV-E2 in the extract (Figure S5).

The 1/*n* parameter of the Freundlich adsorption model might provide a preliminary insight into the adsorption process. The adsorption is favorable if 1/*n* < 1, linear for 1/*n* = 1, and unfavorable if 1/*n* > 1 [62]. For intact sorbent (CV), the adsorption might be classified as linear or slightly unfavorable (1/*n* = 1.1), whereas favorable adsorption was reported for each sorbent with extracted lipids (CV-E2, CV-E3). For sorbent treated with ultrasound (CV-E1), 1/*n* = 1.8 suggested unfavorable adsorption, most probably as a result of destroying the algae cells that caused an increased heterogeneity of the sorbent particles. Compared with other sorbents, CV-E1 was also characterized by a 2-fold lower value of heat of adsorption estimated from the Temkin model (*b_T_* = 7.8 vs. *b_T_* > 13.0 kJ/mol), proving the relative unfavourability of the DCF adsorption onto this material.

The energy of adsorption (*E_DR_*) was estimated for each sorbent using the Dubinin–Radushkevich model. Physisorption is characteristic for *E_DR_* < 8 kJ/mol, whereas chemisorption for *E_DR_* is between 8 and 16 kJ/mol [63]. The *E_DR_* values obtained for investigated sorbents varied in the range of 2.6–3.8 kJ/mol, characteristic of physisorption. Moreover, similar values of *E_DR_* (~3.0 kJ/mol) were reported by Hifney et al., who investigated the adsorption of DCF using living cells of *Chlorella* sp. [22]. Thus, it might be assumed that modifications of intact *Chlorella vulgaris* material, proposed in this work, did not affect the mechanism of adsorption significantly in terms of Dubinin–Radushkevich model assumptions.

The influence of lipid presence in the investigated sorbents on the DCF adsorption was reflected in the calculated mean distribution coefficients (*K_D_*) (Table 2). The *K_D_* value for intact *Chlorella vulgaris* sorbent (CV, *K_D_* = 2.3) was almost the same as for the sample extract with dominant lipid fraction (CV-E4, *K_D_* = 2.6). This observation suggested that DCF adsorption onto intact sorbent might result from the partition of drug molecules between the solution and lipid fraction of the algae. The extraction process, used in the case of CV-E2 and CV-E3 sorbents, caused a significant decrease in *K_D_* values (from 2.3 to 1.1 and 0.7, respectively) proving the important role of lipids in the adsorption of DCF onto the algae.

Taking into account the obtained results and the theoretical assumptions of the used adsorption models, the DCF sorption onto dried *Chlorella vulgaris* might be considered as a complex process. However, based on the calculated adsorption energies (*E_DR_* = 2.6–3.8 kJ/mol), physical sorption seems to be predominant, consistent with the rapid sorption kinetics and reversible, pH-switchable desorption observed experimentally. We found that DCF sorption is governed by equilibrial speciation of the drug between solution and dried *Chlorella vulgaris* biomass as a result of the preferred DCF affinity to the lipid fraction of biomass. DCF is a weak acid (pK_a_ ≈ 4.0–4.2); thus, in the sorption medium (HCl-KCl buffer, pH = 2.0), it occurs in an undissociated form that is more hydrophobic and characterized by high affinity for the lipid fraction of biomass, according to the principle “like dissolves like”. When the pH of the medium is higher than that of the drug pK_a_, the DCF dissociated (hydrophilic) form is dominant, with higher affinity to the polar solvent rather than to the lipid fraction of biomass. This phenomenon explains the inverse relationship between sorption efficiency and pH and indicates the key role of hydrophobic interactions and partitioning during DCF sorption. This interpretation is supported by similar distribution coefficients obtained for intact biomass and the lipid-rich extract (CV-E4). In contrast, lipid extraction from biomass led to a marked decrease in values of distribution coefficients, confirming the importance of lipids in DCF sorption onto dried *Chlorella vulgaris* biomass. In addition to lipid-mediated partitioning, surface adsorption of DCF via interaction with functional groups present in the cell wall of dried biomass (hydroxyl, carboxyl, and amine groups confirmed by ATR-FTIR, Figure 4), as well as filling of pores (obtained after lipid extraction and visible in SEM micrographs, Figure 1), might also contribute to the overall sorption process.

#### 2.2.3. Maximum Sorption Capacity of Sorbents

**Figure 6:**
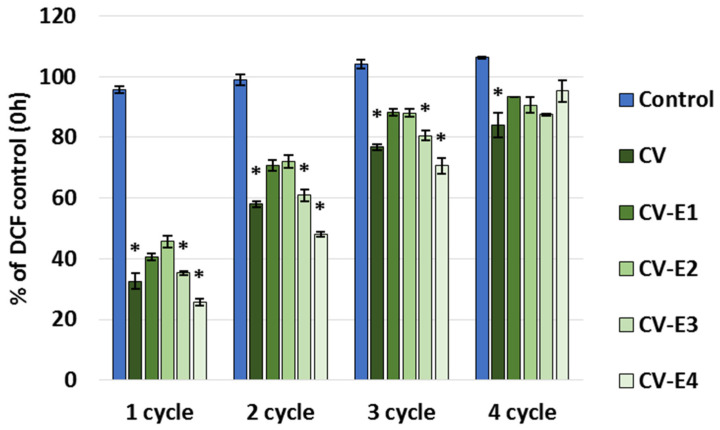
Part of DCF remaining in the supernatant (% of control) after adding DCF solution (1 mg/L, pH 2.0) to the tested sorbents. Each cycle included the addition of DCF to the sorbent in buffer, stirring for 24 h, centrifugation, sampling, discarding the remaining supernatant, and adding new portions of buffer and DCF; cycles were repeated until saturation of the sorbents was achieved. Values represent mean ± SEM; *—significant difference compared to the control at *p* < 0.05 (Kruskal–Wallis, post hoc Dunn’s test).

The maximum sorption capacity study revealed that all the studied sorbents continue to bind DCF when new portions of DCF are added, and after the fourth sorption cycle, they became saturated with the DCF (Figure 6). This can be explained by the fact that we used a low concentration of DCF (1 mg/L), and after its sorption and establishment of equilibrium, its concentration in the solution decreased noticeably, lowering the driving force for DCF sorption, which, as is known, is directly dependent on its initial concentration [5,6,7,8].

The total sorption capacity (calculated based on the summation of DCF bound to the sorbent in each cycle) was the highest for CV-E4 (3.16 ± 0.02 mg/g). The intact *Chlorella vulgaris* (CV) exhibited the highest sorption capacity (3.01 ± 0.09 mg/g), which slightly decreased after sonication (CV-E1: 2.78 ± 0.10 mg/g), and was significantly reduced as the lipid content decreased—to 2.24 ± 0.12 mg/g in the CV-E2 group (*p* = 0.029 vs. CV) and to 2.13 ± 0.12 mg/g in the CV-E3 group (*p* = 0.007 vs. CV). It is worth noting that the DCF concentration–cycle relationship for CV-E4 was almost linear (*R*^2^ = 0.9995) with the addition of new portions of DCF in new cycles. Other studies have also shown that the presence or absence of lipids in sorbents causes important changes in their sorption behavior and that removal of lipids by solvent extraction generally increases the nonlinearity of the adsorption isotherm when using soils [64,65,66] or lipid-reach microalgae after lipid extraction as sorbents (experimental results were fitted better with the Langmuir than the Freundlich isotherm model) [67]. It is worth noting that in these works, after the extraction of lipids, the sorption affinity for the hydrophobic organic compounds, polycyclic aromatic hydrocarbons, and solutes capable of polar interaction increased. At the same time, reports on the sorption capacity of dry *Chlorella* sp. biomass after lipid extraction varies: in the case of tetracycline, it was higher than that of the intact material [57], and in the case of cephalexin, a decrease in absorption by the extracted *Chlorella* biomass was shown [58]. In both cases, a two-step extraction process was used to prepare the sorbent, including sonication followed by Soxhlet extraction; however, the conditions were different, which could affect the results.

#### 2.2.4. Dynamics of the Sorption/Desorption Processes

In the preliminary experiments to determine the time required for sorption, it was found that when large doses of sorbent are used, sorption occurs quickly and equilibrium is established within the first 30 min, and when using lower concentrations of the sorbent, maximum sorption is observed within an hour (Figure S3). Thus, the instantaneous nature of the binding of DCF (90% in the first minute) does not allow the use of kinetic equations; however, during these experiments, a 24 h contact time was applied to ensure complete and reproducible equilibrium, in accordance with literature data. A similar pattern was shown by Larous et al. when, at the same concentration of sorbent (carbon prepared from olive stones), the removal of DCF occurred in a matter of minutes at pH 2.0 and increased with increasing pH to 10 [7]. Jodeh et al. also demonstrated a rapid increase in sorption amount during the first 15 min [6]. Baccar et al. showed rapid sorption of DCF (within 5 min) by activated carbon at pH 4.1 [68]. The desorption kinetics of DCF when changing pH from 2.0 to 9.0 was also rapid, reaching equilibrium within 10 min (Figure S4), which highlights the practical advantages of the sorbent—enabling both fast and efficient sorption and equally fast regeneration through desorption. In addition, an important parameter influencing the sorption of DCF is its concentration: for DCF solutions with higher initial concentrations, a longer time to establish equilibrium was required [7].

#### 2.2.5. Desorption Studies

Preliminary studies show that desorption of DCF from saturated CV occurs quickly after buffer replacement (pH 2.0 to pH 9.0) (Figure S4a). The same short time was required for the desorption of bound DCF back into the solution when the pH was adjusted to 9.0, 11.0, and 12.5 (Figure S4b); also, at pH 11.0 and 12.5, hydrolysis of the sorbent is observed after 24 h, which makes it is unsuitable for reuse (the hydrolysis was assessed visually by the turbidity of the solution). At pH 9.0, the sorbent retained its binding properties after at least one sorption/desorption cycle. To test this, 88.4% of DCF was sorbed in pH 2.0 buffer (*Chlorella* 5 g/L, DCF 1 mg/L). Then 93.9% was desorbed by pH 9.0 buffer. The sample was centrifuged, fresh DCF (1 mg/L in pH 2.0) was added to the sediment, and the sorption occurred again and reached 84%. The cyclic sorption–desorption experiment comprising five regeneration cycles did not reveal any statistically significant differences in either DCF sorption or desorption efficiency between cycles. On average, 63.5 ± 1.11% of diclofenac was adsorbed over the five cycles, of which 99.4 ± 5.4% was subsequently desorbed. These results indicate long-term structural stability of the biomass, as well as the preservation of its sorption properties during repeated use.

Regardless of the procedure of regeneration, the desorption of DCF with a change in the solution pH from 2.0 to 9.0 showed that the degree of recovery in each case was more than 90%, demonstrating the sorbent’s potential for efficient and repeated use in practical applications (Table 3). There was a significant decrease in the recovery rate in the CV-E1 group compared to the control, which can be explained by a stronger binding of DCF to the lipids released after sonication.

### 2.3. Study Strengths and Limitations

The major strength of the study lies in its systematic, mechanism-oriented evaluation of diclofenac sorption on dried *Chlorella vulgaris* biomass, including intact material and its modified forms. As summarized in Table 4, *Chlorella* biomass demonstrates sorption capacities comparable to several reported biobased sorbents operating under similar concentration ranges, while requiring substantially less complex and less energy-consuming preparation than many alternative materials, such as chemically activated biochars, nanocellulose-based sorbents, or dendrimer-coated silica.

At the same time, Table 4 also clearly illustrates the limitations of the proposed biosorbent. Although reusable and structurally tunable, the maximum sorption capacity of *Chlorella* biomass remains lower than that of activated carbons, nanocellulose, or biochar-based materials prepared via energy-intensive thermal or chemical activation. Moreover, other limitations should also be acknowledged. The experiments were conducted under controlled laboratory conditions using buffered solutions and diclofenac concentrations higher than those typically measured in real water matrices, limiting direct extrapolation to practical applications. Sorption efficiency was strongly dependent on acidic pH, which may constrain implementation without pH adjustment. It should be noted, however, that from a practical perspective, reducing the pH to approximately 4 (see Figure S1) may already be sufficient to achieve effective sorption, representing a considerably simpler and more cost-effective approach for real-world applications.

Future work should address scalability aspects in both quantitative terms (e.g., application in wastewater treatment plants, biomass availability, and regeneration under continuous conditions) and qualitative terms, including the applicability of the sorbent to a broader range of target compounds beyond diclofenac.

## 3. Materials and Methods

### 3.1. Chemicals

Acetonitrile (CAS: 75-05-8), methanol (CAS: 67-56-1), hexane (CAS: 110-54-3), chloroform (CAS: 67-66-3), formic acid (CAS: 64-18-6), acetic acid (CAS: 64-17-7), diclofenac sodium (CAS: 15307-79-6), sodium phosphate monobasic (CAS: 7558-80-7), and Nile red (CAS: 7385-67-3) were purchased from Sigma Chemical Company (St. Louis, MO, USA). Di-sodium hydrogen phosphate dodecahydrate (CAS: 10039-32-4), sodium hydroxide (CAS: 1310-73-2), potassium hydroxide (CAS: 1310-58-3), and hydrochloric acid (CAS: 7647-01-0, POCH) were from POCH (Gliwice, Poland). Potassium phosphate monobasic (CAS: 7778-77-0) was from Chempur (Piekary Śląskie, Poland). Analytical-grade water for mobile phase preparation was obtained from Hydrolab (Wislina, Poland) Milli-Q water purification system.

### 3.2. Selection of Sorbent and Its Modification

*Chlorella vulgaris* (CV) powders of commercial origin obtained from organic farming, with certificates confirming their compliance with European Union standards (manufacturer: BATOM.PL, Krakow, Poland, Certificate number: PL-EKO-03.616-0000567.2024.002), was used. Characteristics of *Chlorella* powder, according to the producer’s certificate, are presented in the Supplementary Materials (Table S1). Three types of sorbent modifications were applied to test the influence of physical properties and chemical composition on the sorption process: ultrasonic treatment, lipid extraction, and their combination (Table S2).

In the first modification (CV-E1), to verify whether the active area of the sorbent plays a role in the sorption process, the powder was dispersed in analytical-grade water and exposed to sonication for 1.5 h to destroy the cell structure. Following this step, the resulting mixture was centrifuged (4000× *g* for 10 min), and the pellet was lyophilized and powdered.

We also aimed to verify the role of cellular lipids in the sorption process, so the second modification (CV-E2) was the extraction of lipids in mild conditions to avoid cell destruction; for this purpose, a Soxhlet extraction with a mixture of methanol:chloroform:hexane (1:1:1, *v*/*v*/*v*) was employed. The third modification (CV-E3) of the sorbent was the simultaneous use of solvent extraction (methanol:chloroform:hexane (1:1:1, *v*/*v*/*v*) and sonication (conditions: weight of biomass = 1.5 g, solvent volume = 10 mL, time per cycle = 10 min, number of cycles = 10). In both cases, the extraction cycles were repeated until the solvent became transparent (free of pigments), and the biomass residue after extraction was lyophilized and ground to obtain a powder. Additionally, lipid fraction isolated by Soxhlet extraction (CV-E4) was also tested for the ability towards DCF removal from aqueous buffers. For this purpose, the extract was concentrated by evaporation in a heating block (at 50 °C), and the residue (lipid fraction) was dispersed in the test buffers using sonication to get a working concentration of 0.5 g/L.

### 3.3. Selection and Preparation of Buffers

Considering the low aqueous solubility of DCF at pH below 2.0, as well as the low sorption at pH 7.0 shown during the preliminary tests, we selected three pH values for buffers (2.0, 4.0, and 6.0) (Table S3) from this range, which made it possible to track the dependence of sorption on pH. In preliminary studies, these three buffers provided a stable pH under conditions simulating sorption (1.5 mg/L DCF, 0.5 g/L *Chlorella*) (Table S4). In addition, alkaline buffer with pH = 9 was used to study desorption (Table S3).

### 3.4. Instrumentation

#### 3.4.1. Microscopical Assessment

The morphology of the sorbents was studied using a LAB 40 series Metallographic Microscope (OPTA-TECH, Warsaw, Poland) equipped with an MI6 camera (OPTA-TECH, Warsaw, Poland) and an OPTA-TECH LMPlan 100×/0.80 ∞/0 WD 2.1 objective, under bright-field conditions (samples were prepared using distilled water). SEM analysis on sorbents was performed using the scanning electron microscopes Prisma E (Thermo Fisher Scientific, Waltham, MA, USA) and Phenom XL (Thermo Fisher Scientific, Waltham, MA, USA). To obtain SEM images, sorbent samples (CV, CV-E1, CV-E2, and CV-E3) were previously lyophilized and were coated with an Au/Pd thin film by sputtering, using the SPI Module Sputter Coater equipment.

#### 3.4.2. Physical and Molecular Characteristics of Sorbents

Fluorescent dye Nile red (NR) was dissolved in DMSO to prepare stock solutions (0.5 g/L). A quantity of 5 μL of the stock solution was added to a 1 mL suspension of sorbents (0.5 g/L) in buffer (pH 2.0). Unstained sorbent samples and dye in the buffer were analyzed as negative controls. After vortex mixing, the samples were incubated at room temperature for 15 min and analyzed on a flow cytometer (Amnis^®^ FlowSight^®^ imaging flow cytometer, AMNIS, Seattle, WA, USA) fitted with 488 nm excitation lasers; fluorescence of the dye was registered at the 560–595 nm band, and autofluorescence of chlorophyll was registered at the 640–745 nm band. At least 1000 gated cells per sample were collected and analyzed; each sample collection was performed three times (three replicates). Data processing, histograms, and graphs of fluorescent and unstained samples were carried out using the IDEAS application (v. 6.2.187.0).

#### 3.4.3. Porosity Measurements

The nitrogen adsorption–desorption isotherms were determined on the ASAP 2420M analyzer (Micromeritics, Norcross, GA, USA). Prior to the measurements, the samples of *Chlorella* materials (CV, CV-E1, CV-E2, and CV-E3) were degassed under vacuum at 50 °C for 24 h. The pore diameter (D_BJH_) was calculated from the desorption branch of the isotherm using the Barrett–Joyner–Halenda (BJH) method. The specific surface area (S_BET_) and pore volume (Vp) were calculated using Brunauer–Emmett–Teller (BET) and BJH methods, respectively.

#### 3.4.4. ATR-FTIR Studies

The Attenuated Total Reflectance-Fourier Transform Infrared Spectroscopy (ATR-FTIR) measurements of *Chlorella* materials (CV, CV-E1, CV-E2, CV-E3, CV-E4) were performed on a Jasco FT/IR-4700 spectrometer equipped with ATR PRO ONE diamond prism accessory (Jasco, Pfungstadt, Germany) in the range of 4000–400 cm^−1^ (32 scans per sample, 4 cm^−1^ resolution).

#### 3.4.5. HPLC–UV

The HPLC system consisted of a Waters 2695 separation module coupled to a Waters 2487 dual λ absorbance detector. The wavelengths used for the UV detector were 215 and 275 nm. Data processing was carried out with Empower 3 software (Waters, MA, USA). Chromatographic conditions: the chromatographic separation was performed using a Zorbax Extend-C18 column (150 × 4.6 mm, 3.5 μm particle size from Agilent Technologies, Santa Clara, CA, USA) at 40 °C. The mobile phase was composed of analytical-grade water (A) acidified with 0.1% (*v*/*v*) formic acid and acetonitrile (B), with gradient elution at a flow rate of 1 mL/min. (Table S5), using an injection volume of 20 µL. A stock solution of DCF (1 g/L) was prepared in methanol and stored at −20 °C. Standard solutions were prepared by diluting stock solution in water in the range of 0.05–50 mg/L to construct a 6-point calibration curve, with a limit of detection and quantification of 0.09 and 0.28 mg/L, respectively (a signal-to-noise ratio of 3:1 was used to estimate the LOD; for LOQ, a ratio of at least 10:1 was used).

### 3.5. Diclofenac Sorption Studies

#### 3.5.1. DCF Solubility

Considering the expected poorer solubility of DCF at low pH, and to prevent DCF loss due to precipitation during the planned experiments, DCF solubility was assessed experimentally at pH 2.0 and was equal to 1.70 ± 0.12 mg/L at room temperature (see Supplementary Materials), so in further experiments, the maximum concentration of DCF did not exceed 1.5 mg/L (see Supplementary Materials).

#### 3.5.2. The Influence of pH on DCF Sorption

The sorption of DCF by selected sorbents (CV, CV-E1, CV-E2, CV-E3, CV-E4) in concentration of 0.5 g/L was studied at pH 2.0, 4.0, and 6.0 buffers (Table S3) by dispersing 7.5 mg of each sorbent in 15 mL of buffer, adding 100 μL of DCF stock solution (150 mg/L) to reach 1 mg/L, and stirring for 24 h, followed by centrifugation and measurement of DCF concentration in the supernatants by HPLC.

#### 3.5.3. The Influence of Sorbent Concentration on DCF Sorption Efficiency

The possibility of achieving a higher DCF removal rate by increasing the sorbent concentration was also tested; for this purpose, *Chlorella* powder was resuspended in DCF solution (pH 2.0, C = 1 mg/L) to obtain concentrations of 1, 2.5, 5, 10, and 20 g/L.

#### 3.5.4. Studies on Sorption Equilibrium State

The dynamics of DCF sorption on *Chlorella vulgaris* powder (CV) were studied by measuring DCF concentration at different times (1, 5, and 30 min) after adding it (1 mg/L) to a high-concentration *Chlorella* suspension (5 g/L) at pH 2.0. In addition, equilibrium time was determined using a low-concentration CV suspension (0.5 g/L) after 1, 24, and 48 h.

#### 3.5.5. The Adsorption Isotherms

To determine the adsorption isotherms, the DCF adsorption studies on four types of *Chlorella vulgaris* sorbents (CV, CV-E1, CV-E2, CV-E3) were carried out. DCF adsorption from aqueous buffer (pH 2.0) was performed as follows: A quantity of 7.5 mg of *Chlorella vulgaris* biomass was weighed into a polypropylene falcon tube and suspended in 15 mL of DCF buffered solution with initial drug concentrations of 0.1, 0.25, 0.5, 1.0, and 1.5 mg/L. As previously mentioned, the highest concentration used (1.5 mg/L) was equal to 88% of drug solubility in the buffer solution pH 2.0 (1.7 mg/L) to prevent spontaneous precipitation of DCF during the adsorption studies. The samples were continuously mixed using a rocker mixer for 24 h at room temperature (60 rpm, Digital Waving Rotator, Thermo Scientific, Waltham, MA, USA) to achieve the equilibrium state. Next, the samples were centrifuged at 10,000 *g* for 10 min, 1 mL of supernatant was transferred to the glass autosampler vial, and the residual concentration of DCF was measured by the HPLC with UV detection. The concentration of DCF in the supernatant was corrected for non-algae-related sorption (reduction in DCF level in control samples without algae, treated under the same conditions) and was used as an indirect measure of sorption efficiency. The adsorption experiments were performed in triplicate.

The total amount of DCF sorbed onto each sorbent and the adsorption efficiency were calculated using Equations (1) and (2), respectively:

(1) $$q_{e}=\frac{\left(C_{0}-C_{e}\right)\cdot V}{m}$$

(2) $${\%}_{Ads}=\frac{C_{0}-C_{e}}{C_{0}}\cdot 100\%$$

where *q_e_*—amount of DCF sorbed per 1 g of sorbent at equilibrium state (mg/g), *C*_0_—initial concentration of DCF solution (mg/L), *C_e_*—the equilibrium concentration of DCF in the solution after adsorption (mg/L), *V*—volume of solution (L), *m*—mass of sorbent (g), %*_Ads_*—adsorption efficiency (%).

The distribution coefficient *K_D_* (Equation (3)) of DCF [75] was calculated for all investigated sorbent modifications (CV, CV-E1, CV-E2, CV-E3), as well as for extracted lipid fraction (CV-E4) for comparative purposes:

(3) $$K_{D}=\frac{q_{e}}{C_{e}}$$

where *q_e_*—the amount of DCF sorbed per 1 g of sorbent at equilibrium state (mg/g), *C_e_*—the equilibrium concentration of DCF in the solution after adsorption (mg/L). The *K_D_* values were calculated from the slope of the plot of *q_e_* versus *C_e_* [76].

In addition, to describe the potential mechanism of DCF sorption onto chosen CV materials, four adsorption models were applied, namely, Langmuir, Freundlich, Dubinin–Radushkevich, and Temkin. To evaluate each model fitting, the coefficient of determination (*R*^2^) was calculated based on the linear forms of adsorption isotherms (Equations (4)–(9)).

The Langmuir model assumes the monolayer adsorption onto the homogenous surface, which can be expressed as [62].

(4) $$\frac{1}{q_{e}}=\frac{1}{q_{L(max)}}+\frac{1}{q_{max}\cdot K_{L}}\cdot \frac{1}{C_{e}}$$

where *q_L_*_(*max*)_—the estimated maximum adsorption capacity (mg/g), *K_L_*—Langmuir constant (L/mg).

The Freundlich model is an empirical model that can be used to describe the multilayer adsorption onto heterogeneous sites as follows [62]:

(5) $$logq_{e}=logK_{F}+\frac{1}{n}logC_{e}$$

where *K_F_*—Freundlich constant [(mg/g)(L/mg)^−1/*n*^], 1/*n*—adsorption intensity coefficient.

The Dubinin–Radushkevich model is based on the theory of micropore volume filling, in which the molecules of sorbate are successively adsorbed inside the micropores rather than forming adsorption layers, and is described by the following equations [77,78]:

(6) $$lnq_{e}=lnq_{DR(max)}-K_{DR}\cdot \varepsilon^{2}$$

(7) $$\varepsilon =R\cdot T\cdot \ln\left(\frac{C_{s}}{C_{e}}\right)$$

where *q_DR_*_(*max*)_—the estimated maximum adsorption capacity (mg/g), *K_DR_*—Dubinin–Radushkevich constant (mol^2^/J^2^), *ε*—Polanyi potential (J/mol), *R*—gas constant [8.31 J/(mol·K)], *T*—temperature (K), *C_s_*—solubility of DCF in a given solvent (mol/L), *C_e_*—equilibrium concentration of DCF after adsorption (mol/L).

From the Dubinin–Radushkevich model, the adsorption energy *E_DR_* (kJ/mol) has been also calculated using the following equation [77,78]:

(8) $$E_{DR}=\frac{1}{1000\cdot \sqrt{{2K}_{DR}}}$$

The Temkin isotherm model considers the influence of indirect interactions between adsorbate molecules on the adsorption process assuming the linear decrease in the heat of adsorption with an increasing sorbent’s surface coverage of sorbent, and can be expressed as [79]

(9) $$\frac{q_{e}}{q_{max}}=\left(\frac{R\cdot T}{b_{T}}\right)\cdot lnK_{T}+\left(\frac{R\cdot T}{b_{T}}\right)\cdot lnC_{e}$$

where *q_max_*—maximum loading of DCF onto adsorbent (mg/g) determined experimentally, *b_T_*—Temkin constant related to the heat of adsorption (J/mol), *K_T_*—the equilibrium binding constant.

#### 3.5.6. Sorption Capacity

Preliminary studies have shown that the use of high concentrations of sorbent (1, 2.5, 5, 10, and 20 g/L) leads to the complete removal of DCF from the solution (Figure S2). However, to calculate *C_e_*, the final concentration of DCF must be greater than zero for each concentration studied, so we chose a sorbent concentration of 0.5 g/L, at which complete sorption did not occur. Based on the above, to more accurately determine the maximum sorption, we used one dose of the sorbent (0.5 g/L), to which DCF (1 mg/L) was added in several cycles until its concentration equilibrium was established, which corresponded to complete saturation of the sorbent (1 cycle lasted 24 h, after which the sorbent was separated by centrifugation and a new portion of DCF solution was added).

#### 3.5.7. Desorption Studies

To test the ability to recover DCF from biomass back into the solution, DCF-saturated algae biomass (obtained in the same way as described in the sorption studies; Section 3.5.2) was incubated with alkaline buffer (pH 9.0). Briefly, after incubation of the sorbents (0.5 g/L) with 15 mL of DCF solution (1 mg/L) in pH 2 buffer using a rocker mixer (60 rpm) at room temperature for 24 h and separated by centrifugation (4000× *g*, 10 min), the supernatant was discarded, and then 15 mL of alkaline buffer was added; the biomass was resuspended and incubated with the buffer in the same manner. Next, the solution was centrifuged (4000× *g*, 10 min), the DCF concentration in the solution was determined, and the recovery was calculated as a percentage of DCF relative to the mass of added DCF (total amount (mg)); in addition, an adjustment was made using control samples without adding sorbents to exclude potential sorption of DCF on the walls of the falcons. Also, to demonstrate the reusability of the sorbent and to assess the possibility of reducing the sorption and desorption times to 5 min, the experiment described above was repeated using the same pellet for five consecutive sorption/desorption cycles, with the mixing time shortened to 5 min in each cycle.

Additionally, to enhance the practical applicability of the sorbents, we tested an alternative regeneration method by adjusting the pH to 9.0 using NaOH solution instead of buffer replacement. Also, to check the regeneration of the sorbent (5 g/L), we carried out 2 sorption-desorption cycles with DCF (1 mg/L) one after another.

#### 3.5.8. Statistical Analysis

Statistical analysis was performed using MS Excel 2013 (Microsoft Inc., Redmond, WA, USA) and Statistica 13.0 (TIBCO Software Inc., Palo Alto, CA, USA). All data were expressed as the means of at least three independent experiments ± SEM (standard error). A post hoc nonparametric Mann–Whitney U test was used for pairwise analysis (*p* < 0.05) for the population that could not be assumed to be normally distributed. As an analog of ANOVA for small sample sizes, the Kruskal–Wallis test was used to determine statistical differences between independent groups (*p* < 0.05). To eliminate the increase in the possibility of a first-order error, a post hoc Dunn’s test for pairwise comparisons between each independent group was also applied.

## 4. Conclusions

This study demonstrates that dried *Chlorella vulgaris* biomass represents a versatile biosorbent material. The results show that diclofenac removal is governed by a mixed, predominantly physical sorption mechanism involving hydrophobic partitioning into lipid domains and surface adsorption onto functional groups of the biomass, with strong dependence on solution pH and biomass composition.

Importantly, the observed pH-switchable and reversible sorption, combined with rapid kinetics and structural stability over multiple regeneration cycles, highlights the potential of *Chlorella* biomass, not only for contaminant removal but also for broader applications where controlled sorption and desorption are required. These include laboratory-scale separation processes, preconcentration and clean-up procedures in analytical chemistry, and the development of bio-based sorptive materials with tailored interaction profiles.

A critical limitation of the present study is that the key sorption experiments yielding the highest efficiencies were conducted under strongly acidic conditions (pH 2), which differ substantially from typical wastewater and surface water matrices. Therefore, the results should be interpreted explicitly as a proof of concept demonstrating mechanistic principles rather than as evidence of direct real-world applicability. Any environmental application would require prior pH adjustment or alternative process configurations, which were beyond the scope of this work.

Although diclofenac was used as a model compound, the underlying sorption mechanisms identified in this work, particularly hydrophobic interactions, surface adsorption, and lipid-mediated partitioning are not specific to this compound alone. Therefore, the findings may be transferable to other organic contaminants and xenobiotics with comparable physicochemical properties. Overall, the results position *Chlorella vulgaris* biomass as a promising and adaptable sorptive material with application potential extending beyond conventional phytoremediation purposes.

## Figures and Tables

**Table 1:** The porosity characteristics of the investigated *Chlorella vulgaris* sorbents.

Sample	S_BET_ (m^2^/g)	D_BJH_ (nm)	Vp (cm^3^/g)
CV	-	-	-
CV-E1	0.39	9.09	0.001
CV-E2	1.48	25.41	0.008
CV-E3	0.56	25.59	0.002

**Table 2:** The calculated parameters from Langmuir, Freundlich, Dubinin–Radushkevich, and Temkin adsorption models, together with distribution constants for the investigated sorbents (CV, CV-E1, CV-E2, and CV-E3) and sample extract (CV-E4).

Parameter	CV	CV-E1	CV-E2	CV-E3	CV-E4
Langmuir					
*q_L_*_(*max*)_	-	-	12.5	-	2.0
*K_L_*	-	-	0.11	-	1.65
*R* ^2^	0.9882	0.6672	0.9986	0.8664	0.9700
Freundlich					
1/*n*	1.1	1.8	0.94	0.77	0.9
*K_F_*	2.3	7.2	1.2	1.1	2.1
*R* ^2^	0.9891	0.8304	0.9844	0.8257	0.9847
Dubinin–Radushkevich					
*E_DR_*	3.8	2.6	3.6	3.8	3.9
10^8^ *K_DR_*	3.4	7.4	3.9	3.5	3.2
*R* ^2^	0.9521	0.8939	0.9790	0.9336	0.8698
*q_DR_*_(*max*)_	1.2	3.9	1.0	1.0	1.5
Temkin					
*b_T_*	19.2	7.8	15.5	19.2	13.1
*K_T_*	22.3	11.8	14.9	23.7	16.6
*R* ^2^	0.8656	0.9924	0.9199	0.9724	0.7544
Distribution coefficient					
*K_D_*	2.3	3.5	1.1	0.7	2.6
*R* ^2^	0.9975	0.9782	0.9255	0.7841	0.9491

**Table 3:** Desorption of DCF from the studied sorbents after changing the pH to 9.0; *—significant difference compared with control at *p* < 0.05 (Kruskal–Wallis, post hoc Dunn’s test). Data on desorption in the lipid fraction (CV-E4) are not presented, since sonication is required to dissolve the sediment (after centrifugation and discarding the supernatant), which may affect the efficiency of desorption.

Regeneration Methods	DCF Desorption Recovery Rate (%)			
	CV	CV-E1	CV-E2	CV-E3
Resuspending pellet at pH 9.0	95.0 ± 2.5	94.4 ± 0.8 *	98.3 ± 1.2	102 ± 1.4
Adjusting the solution to pH 9.0	98.3 ± 0.4	98.3 ± 0.6	100.9 ± 0.5	101.3 ± 1.1

**Table 4:** Diclofenac sorption performance of *Chlorella vulgaris* dried biomass in comparison with other sorbents from representative studies (2020–2025).

Sorbent [Ref.]	Experimental Conditions	Sorption Performance (Sorption Capacity and/or Removal Efficiency)	Preparation, Scalability and Remarks
*Chlorella vulgaris* biomass (intact and modified) [This study]	Aqueous (spiked), dSPE; 0.1–1.5 mg/L	Sorption capacities (mg/g): CV 3.01, CV-E1 2.78, CV-E2 2.24, CV-E3 2.13; 88.4% removal (CV, 5 g/L); 99% desorption at pH 9	Dry biomass; ultrasound (CV-E1), lipid extraction (CV-E2), ultrasound-assisted solvent extraction (CV-E3). Low–moderate energy; valorization potential (biofuel waste). Reusable (pH-switch).
Silica coated with dendrimeric MA-BDDE copolymer [69]	Surface water (Brda and Vistula rivers), dSPE; 1 µg/mL	Sorption capacity: 2.253 mg/g (MA-BDDE); 0.10 mg/g (Silica Gel 60)	Multi-layer dendrimeric polymer coating (5 layers). High energy/synthesis effort. Reusable. Analytical HPLC-UV/Vis; FT-IR, ^13^C-NMR.
Chemically activated *Microcystis aeruginosa* biomass [12]	Aqueous (spiked), batch adsorption/dSPE; 1–10 mg/L	Sorption capacity: 11.55 mg/g; >93% removal; desorption 87%	KOH and HCl pretreatments (activation). Moderate energy. Reusable. Non-toxic strain ACCMU-118.
Grass nanocellulose (*Cyperus rotundus*) [70]	Aqueous (spiked), dSPE; 50–250 mg/L	*q_max_* 192.3 mg/g (Halsey); *q_e_* 110.9 mg/g (kinetics)	Multistep chemical processing (ethanol/NaOH/H_2_O_2_/H_2_SO_4_), ultrasonication, lyophilization. Very high energy/complexity. Reusability: n/a.
Activated carbon (coconut shell; H_3_PO_4_-activated) [71]	Aqueous (spiked), dSPE; 20–200 mg/L	*q_max_* 166.7 mg/g (Langmuir); *q_e_* 170.3 mg/g (kinetics); >98% removal	Impregnation + heating; washing/drying; size 0.07–0.2 mm; H_3_PO_4_ activation. High energy. Reusability: n/a.
Soybean hulls (chemically and thermally modified) [72]	Aqueous (spiked), batch adsorption/dSPE; 50 mg/L	*q_e_* 17.27 mg/g (kinetics); *q_max_* 96.88 mg/g (Sips)	Chemical treatment + thermal carbonization (muffle furnace), sieving 425–600 µm. Moderate energy. Fast kinetics (~180 min). Low-cost agro-waste.
Low-cost lignite activated cokes (LAC-1, LAC-2) [73]	Aqueous (spiked), batch adsorption/dSPE; 1–100 mg/L	*q_t_* 25 mg/g (LAC-1) and 62 mg/g (LAC-2); 80% (LAC-1) and 85% (LAC-2) removal; ~80% after 3 cycles	Different activation regimes (>700 °C for LAC-1; <300 °C for LAC-2). Moderate–high energy. Reusable (3 cycles).
PVDF flat-sheet membranes [74]	Deionized water (spiked); UVA-assisted; 10–30 mg/L	54% removal after 18 h UVA with PVDF; improved transformation/reduction vs. control	Commercial PVDF discs; ethanol pre-wet + rinse. Moderate energy (UVA). Reusable. Photochemical effect dominates.
Phosphate-modified *Moringa oleifera* seed powder biochar [8]	Aqueous (spiked), batch adsorption/dSPE; 2.5–70 mg/L	95.38 mg/g (kinetics); *q_max_* 100.88 mg/g (Sips); 83% removal	Pyrolysis 450 °C (N_2_) + 0.5 M H_3_PO_4_ treatment; washing to neutral pH; drying. High energy. Recyclable.

Abbreviations: dSPE, dispersive solid-phase extraction; *q_max_*, maximum adsorption capacity derived from equilibrium isotherm models; *q_e_*, adsorption capacity at equilibrium; *q_t_*, adsorption capacity at time *t* obtained from kinetic modeling; CV, intact *Chlorella vulgaris* biomass; CV-E1, ultrasonically treated *Chlorella vulgaris* biomass; CV-E2, lipid-extracted *Chlorella vulgaris* biomass; CV-E3, ultrasound-assisted solvent-extracted *Chlorella vulgaris* biomass; MA-BDDE, dendrimeric copolymer of methylamine and 1,4-butanediol diglycidyl ether; n/a, information is not available; LAC-1, lignite activated coke prepared at >700 °C; LAC-2, lignite-activated coke prepared at <300 °C; PVDF, poly(vinylidene difluoride); UVA, ultraviolet A irradiation.

## Data Availability

The original contributions presented in this study are included in the article/Supplementary Materials. Further inquiries can be directed to the corresponding authors.

## Supplementary Materials

The following supporting information can be downloaded at: https://www.mdpi.com/article/10.3390/ijms27031413/s1. References [5,6,7,8,22,59,68] are cited in the Supplementary Materials.

## Abbreviations

The following abbreviations are used in this manuscript:

NSAIDs	Nonsteroidal anti-inflammatory drugs
DCF	Diclofenac
CV	*Chlorella vulgaris* powder
CV-E1	*Chlorella vulgaris* powder after ultrasonic treatment
CV-E2	*Chlorella vulgaris* powder after lipid extraction (Soxhlet extraction)
CV-E3	*Chlorella vulgaris* powder after ultrasound-assisted solvent extraction
CV-E4	lipid fraction of *Chlorella vulgaris* isolated by Soxhlet extraction
SEM	Scanning electron microscopy
NR	Nile red
D_BJH_	Pore diameter
BJH	Barrett–Joyner–Halenda method
S_BET_	Specific surface area
Vp	Pore volume
BET	Brunauer–Emmett–Teller method
ATR-FTIR	Attenuated Total Reflectance–Fourier Transform Infrared Spectroscopy
LOD	Limit of detection
LOQ	Limit of quantification
*q_e_*	Amount of DCF sorbed per 1 g of sorbent at equilibrium state
*C*_0_	Initial concentration of DCF solution
*C_e_*	Equilibrium concentration of DCF in the solution after sorption
%Ads	Adsorption efficiency
*K_D_*	Distribution coefficient
*q_L(max)_*	Estimated maximum adsorption capacity (Langmuir model)
*K_L_*	Langmuir constant
*K_F_*	Freundlich constant
1/*n*	Adsorption intensity coefficient (Freundlich model)
*q_DR(max)_*	Estimated maximum adsorption capacity (Dubinin–Radushkevich model)
*K_DR_*	Dubinin–Radushkevich constant
*ε*	Polanyi potential
R	Gas constant
T	Temperature
*C_s_*	Solubility of DCF in a given solvent
*E_DR_*	Adsorption energy (Dubinin–Radushkevich model)
*q_max_*	Maximum loading of DCF onto sorbent determined experimentally
*b_T_*	Temkin constant related to the heat of adsorption
*K_T_*	Equilibrium binding constant (Temkin model)
dSPE	Dispersive solid phase extraction
