# Supplementary material for: Investigations of Diclofenac Sorption on Intact and Modified Chlorella vulgaris Biomass with pH-Switchable Desorption
Source: Int J Mol Sci. 2026 Jan 30;27(3):1413. doi: 10.3390/ijms27031413 (PMC12898283; doi:10.3390/ijms27031413)
Supplement: Supplementary file 1 [file ijms-27-01413-s001.zip › ijms-4042697-supplementary.pdf]

Supplementary material for

# Investigations of diclofenac sorption on intact and modified Chlorella vulgaris biomass with pH-switchable desorption

Ivan Liakh [1], Adrian Szewczyk [2], Magdalena Prokopowicz [2], Magdalena Narajczyk [3], Anna Aksmann [4], Darya Harshkova [4], Bartosz Wielgomas [1,*]

[1] Department of Toxicology, Faculty of Pharmacy, Medical University of Gdańsk, al. Gen. Hallera 107, 80-416 Gdansk, Poland; ivan.liakh@gumed.edu.pl (I.L.), bartosz.wielgomas@gumed.edu.pl (B.W.)
[2] Department of Physical Chemistry, Faculty of Pharmacy, Medical University of Gdańsk, al. Gen. Hallera 107, 80-416 Gdansk, Poland; adrian.szewczyk@gumed.edu.pl (A.S.)
[3] Bioimaging Laboratory, Faculty of Biology, University of Gdańsk, ul. Wita Stwosza 59, 80-308 Gdansk, Poland; magdalena.narajczyk@ug.edu.pl (M.N.)
[4] Department of Plant Experimental Biology and Biotechnology, Faculty of Biology, University of Gdańsk, ul. Wita Stwosza 59, 80-308 Gdansk, Poland; anna.aksmann@ug.edu.pl (A.A.), darya.harshkova@ug.edu.pl (D.H.)
* Correspondence: bartosz.wielgomas@gumed.edu.pl

**Table S1.** Characteristics of *Chlorella* powder according to the manufacturer's information.

| **Net weight** | **100 g** |
|---|---|
| **Ingredients** | Organic alga *Chlorella vulgaris* 100% |
| **Nutritional values per 100 g of product** | |
| **Energy value** | 1369 kJ/326 kcal |
| **Fat** | 12 g including saturated fatty acids 1.0 g |
| **Carbohydrates** | 17 g including sugars 0.8 g |
| **Fiber** | 12 g |
| **Protein** | 58 g |
| **Salt** | 1.3 g |

**Table S2.** Description of *Chlorella vulgaris* sorbent modifications and processing conditions.

| **Modification code** | **Modification** | **Processing description** |
|---|---|---|
| **CV-E1** | Ultrasonic treatment (cell disruption) | The biomass was dispersed in analytical-grade water (1.5 g of biomass per 10 mL of water) and sonicated for 1.5 h to destroy cell structure. The biomass was centrifuged (4000 g, 10 min), lyophilized and ground. |
| **CV-E2** | Soxhlet extraction (solvent extraction without cell disruption) | The biomass was subjected to lipid extraction in a Soxhlet apparatus with a methanol:chloroform:hexane mixture (1:1:1, v/v/v) under mild conditions to avoid cell disruption. Extraction was continued until the solvent became colorless. The biomass was lyophilized and ground. |
| **CV-E3** | Ultrasound-assisted solvent extraction (solvent extraction with cell disruption) | The biomass was subjected to ultrasonic treatment (10 cycles of 10 min; 1.5 g of biomass in 10 mL of solvent) in a (methanol:chloroform:hexane mixture (1:1:1, v/v/v) until the solvent was almost colorless. The biomass was lyophilized and ground. |
| **CV-E4** | Isolated lipid fraction | Lipid extract obtained by Soxhlet extraction (as described in CV-E2), was concentrated by evaporation at 50 °C. The resulting residue was dispersed in a buffer solution by ultrasonication to a working concentration 0.5 g/L |

**Table S3.** Recipes for buffers used in the study.

| Sorption studies | | | Desorption studies |
|---|---|---|---|
| **Hydrochloric acid-potassium chloride buffer (88 mM, pH 2.0)** | **Phosphate buffer (14 mM, pH 4.0)** | **Phosphate buffer (44 mM, pH 6.0)** | **Phosphate buffer (128 mM, pH 9.0)** |
| KCl = 6.57 g;<br>HCl = 119 mL;<br>$H_2O$ up to 1 L. | $Na_2HPO_4x12H_2O$ = 5.04 g;<br>$KH_2PO_4$ = 3.01 g;<br>$CH_3COOH$ (glac.);<br>$H_2O$ up to 1 L. | $NaH_2PO_4$ (anh.) = 5.23 g;<br>NaOH (conc.);<br>$H_2O$ up to 1 L. | $KH_2PO_4$ = 17.4 g;<br>KOH (1 mol/L);<br>$H_2O$ up to 1 L. |

**Table S4.** The stability of buffers (pH 2.0, 4.0, 6.0 and 9.0) after 24 hours of exposure to diclofenac (DCF) and *Chlorella* powder, data are present as mean ± SEM. *Chlorella* powder (0.5 g/L) and DCF (1.5 mg/L) were added to buffers (Table S3) and incubated for 24 hours at room temperature on a rocker platform, followed by pH measurement.

| | Control | CV | CV-E1 | CV-E2 | CV-E3 | CV-E4 |
|---|---|---|---|---|---|---|
| **pH2 at 0h** | 2.04±0.003 | 2.03±0.003 | 2.03±0.003 | 2.03±0.003 | 2.03±0.003 | 2.03±0.003 |
| **pH4 at 0h** | 4.04±0.006 | 4.05±0.003 | 4.06±0.003 | 4.05±0.01 | 4.06±0.003 | 4.04±0.007 |
| **pH6 at 0h** | 6.06±0.009 | 6.11±0.013 | 6.12±0.035 | 6.17±0.01 | 6.18±0.006 | 6.07±0.033 |
| **pH9 at 0h** | 9.07±0.003 | 9.01±0.009 | 9.02±0.003 | 9.03±0.003 | 9.01±0.012 | 9.02±0.003 |
| **pH2 after 24h** | 2.03±0.003 | 2.04±0.003 | 2.03±0.003 | 2.03±0.003 | 2.03±0.003 | 2.03±0.003 |
| **pH4 after 24h** | 4.03±0.003 | 4.04±0.003 | 4.06±0.009 | 4.04±0.007 | 4.03±0.003 | 4.04±0.006 |
| **pH6 after 24h** | 6.10±0.006 | 6.14±0.003 | 6.20±0.006 | 6.17±0.012 | 6.14±0.003 | 6.11±0.013 |
| **pH9 after 24h** | 9.06±0.006 | 9.00±0.006 | 9.01±0.003 | 9.01±0.006 | 9.00±0.003 | 9.00±0.009 |

**Table S5.** Gradient program used for DCF separation (where B is acetonitrile).

| Step | Time (min) | Flow (mL/min) | % of B |
|---|---|---|---|
| **1** | 0 | 1 | 40 |
| **2** | 2 | 1 | 40 |
| **3** | 5 | 1 | 90 |
| **4** | 6 | 1 | 90 |
| **5** | 6.5 | 1 | 40 |
| **6** | 10 | 1 | 40 |

**Preliminary studies**

**Testing the Solubility of DCF at Low pH**

Given that the solubility of DCF as a weak acid decreases with decreasing pH, we tested the solubility of DCF at pH 2.0 (the lowest pH of the buffer we used) to prevent DCF loss due to precipitation during the planned experiments. For this purpose, the aqueous suspension of DCF was prepared, adjusted to pH 2.0, and incubated at room temperature with continuous stirring for 24 h. Then, the suspension was filtered through PTFE filters with a pore size of 0.22 μm to remove any undissolved particles, and the concentration of saturated solution was determined by HPLC. The estimated solubility of DCF at pH 2.0 was 1.70 ± 0.12 mg/L (at 19 ± 1 ºC), therefore, to avoid precipitation of DCF, the maximum concentration of DCF used in our study did not exceed 1.5 mg/L.

**The effect of pH on the DCF sorption from solution**

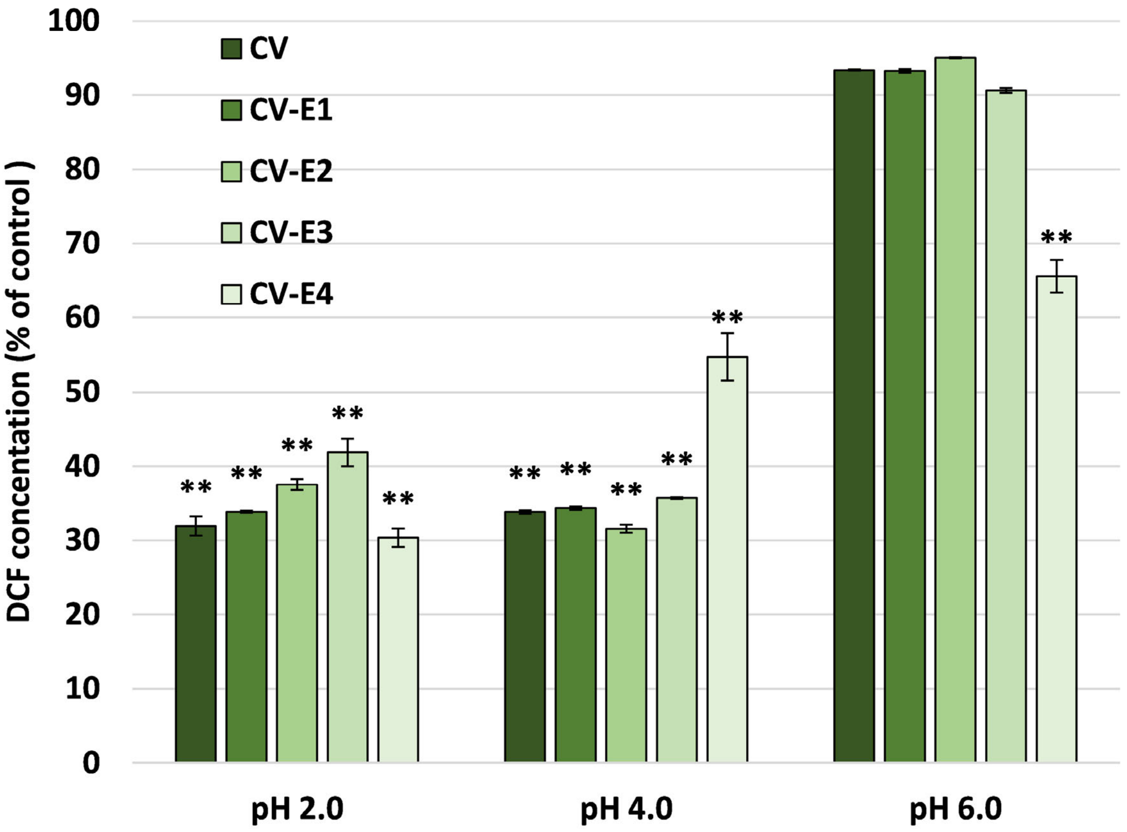


**Figure S1.** Sorption of DCF by selected sorbents at different pH values of the medium: the sorbents (CV, CV-E1, CV-E2, CV-E3 and CV-E4) were weighed (7.5 mg) into 15 mL Falcon-type test tubes and dispersed in 15 mL buffers of pH 2.0, 4.0 and 6.0 to obtain a concentration of 0.5 g/L. Then, 100 μL of DCF stock solution (150 mg/L) was added to obtain a final concentration of 1 mg/L. Then, the mixture was stirred on a rocker platform at room temperature for 24 hours and DCF concentrations were measured in the supernatants obtained after centrifugation (10,000 g, 10 min). DCF in a buffer without the addition of algae after 24 h incubation served as a reference. All experiments were repeated three times. Values are the mean ± SEM; **- significant difference compared with control at $p < 0.01$ (Mann-Whitney U test).

As can be seen in Fig. S1, at pH 6.0, practically no sorption was observed in all sorbents (the concentrations of DCF did not differ from the control ones) except CV-E4. On the contrary in more acidic buffers (pH 2.0 and 4.0), a noticeable decrease in the concentration of DCF occurred, while the difference

between pH 2.0 and pH 4.0 was insignificant (except for CV-E4, which more linearly followed the decrease in pH). Our data are consistent with other studies in which the degree of removal of DCF by different sorbents increases as the pH decreases [1–4]. Although other authors observed maximum sorption at higher pH, which may be due to the nature of the sorbents and their modifications [5], or due to the use of live algae as a sorbent [6]. As noted earlier, the mechanism for this phenomenon may involve the transition of DCF to a neutral form at a pH below pKa, with the van der Waals interaction between DCF and the sorbent surface being enhanced by the process of physical adsorption [3,7]. However, one should take into account the $pH_{PZC}$ of the sorbent, which, at high pH values, can take on a negative charge and enhance electrostatic repulsions [4].

**Effect of Sorbent Dose**

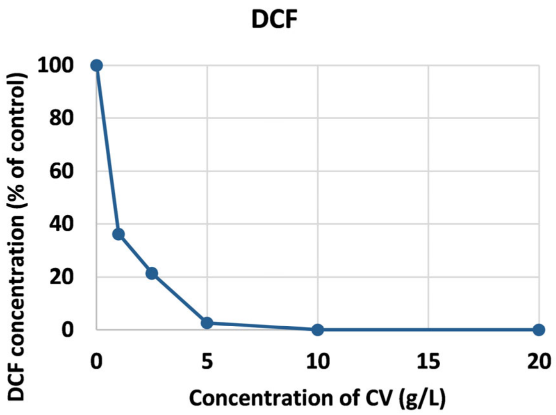


**Figure S2.** The influence of *Chlorella* biomass (CV) concentration on DCF removal from aqueous buffer at pH 2.0. For the test, solutions with biomass concentrations of 1.0, 2.5, 5.0, 10 and 20 g/L were used, and pH was reduced to 2.0, after which the DCF (1 mg/L) was added and stirred for 5 minutes, after filtration the concentrations were determined by HPLC, DCF in a buffer without the addition of algae served as a reference.

**Dynamics of Sorption and Desorption Processes**

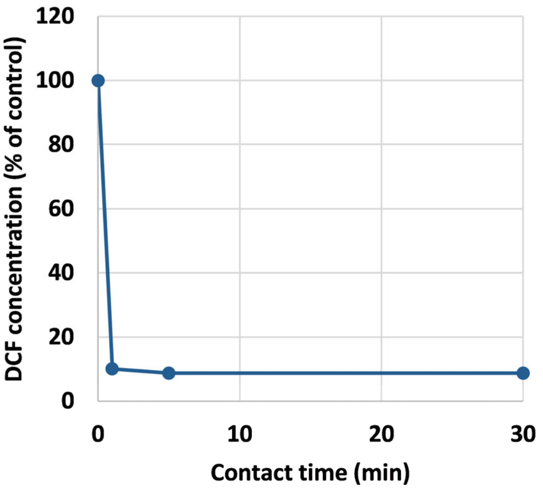


(**a**)

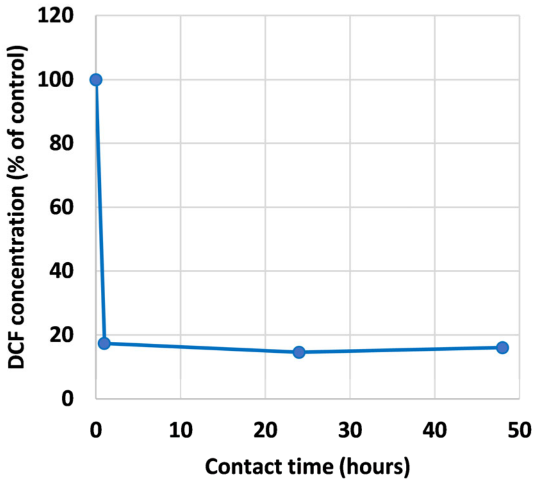


(**b**)

**Figure S3.** Sorption dynamics of DCF on *Chlorella* powder (CV) sorbents: (**a**) - time-dependent DCF sorption in a high-concentration *Chlorella* solution (5 g/L), with DCF concentration measured at 1, 5, and 30 minutes to determine the equilibrium time; (**b**) - comparison of DCF peak areas after 1, 24, and 48 hours in a low-concentration *Chlorella* solution (0.5 g/L). DCF in a buffer without the addition of algae served as a control.

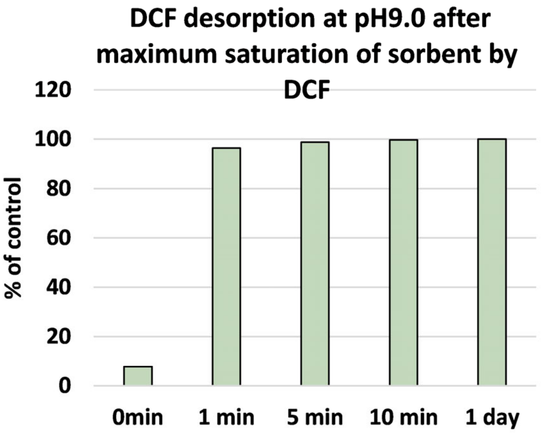


(**a**)

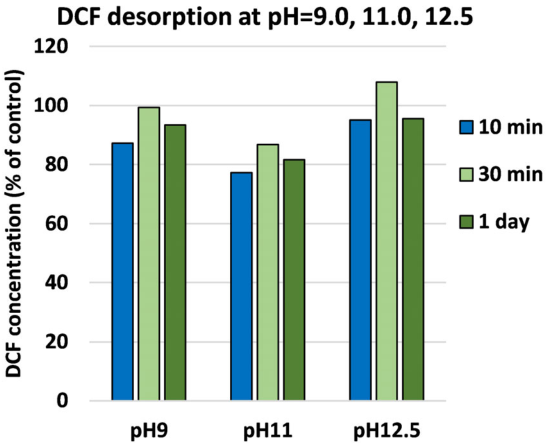


(**b**)

**Figure S4.** Dynamics of DCF desorption from *Chlorella vulgaris* powder (CV): (**a**) - DCF desorption after sorbent saturation (0.5 g/L) with DCF (1 mg/L), followed by buffer replacement (pH 2.0 to pH 9.0), and subsequent concentration measurements; (**b**) - time- and pH-dependent desorption of DCF after 24-hour sorption (*Chlorella* concentration: 0.5 g/L; DCF concentration: 1 mg/L) and subsequent pH adjustment with 5M NaOH. DCF in a buffer without the addition of algae served as a control.

**Microscopy and Flow Cytometry Images**

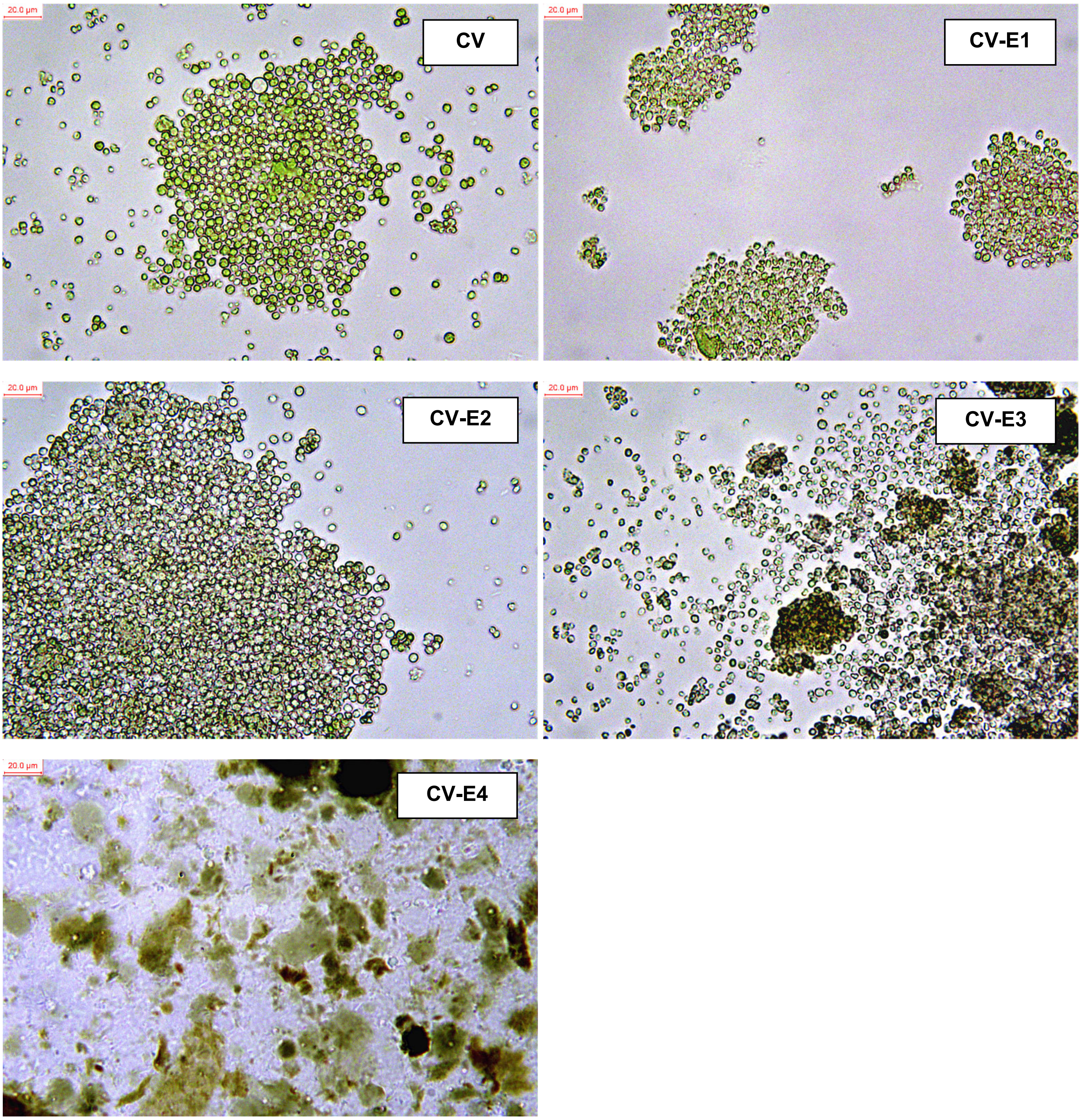


**Figure S5.** Images of *Chlorella vulgaris* powder (CV), *Chlorella vulgaris* powder after sonication (CV-E1), *Chlorella vulgaris* powder after Soxhlet extraction (CV-E2) and *Chlorella vulgaris* powder after ultrasound-assisted solvent extraction (CV-E3) and lipid fraction (CV-E4) diluted in water obtained using light microscopy (magnification x100).

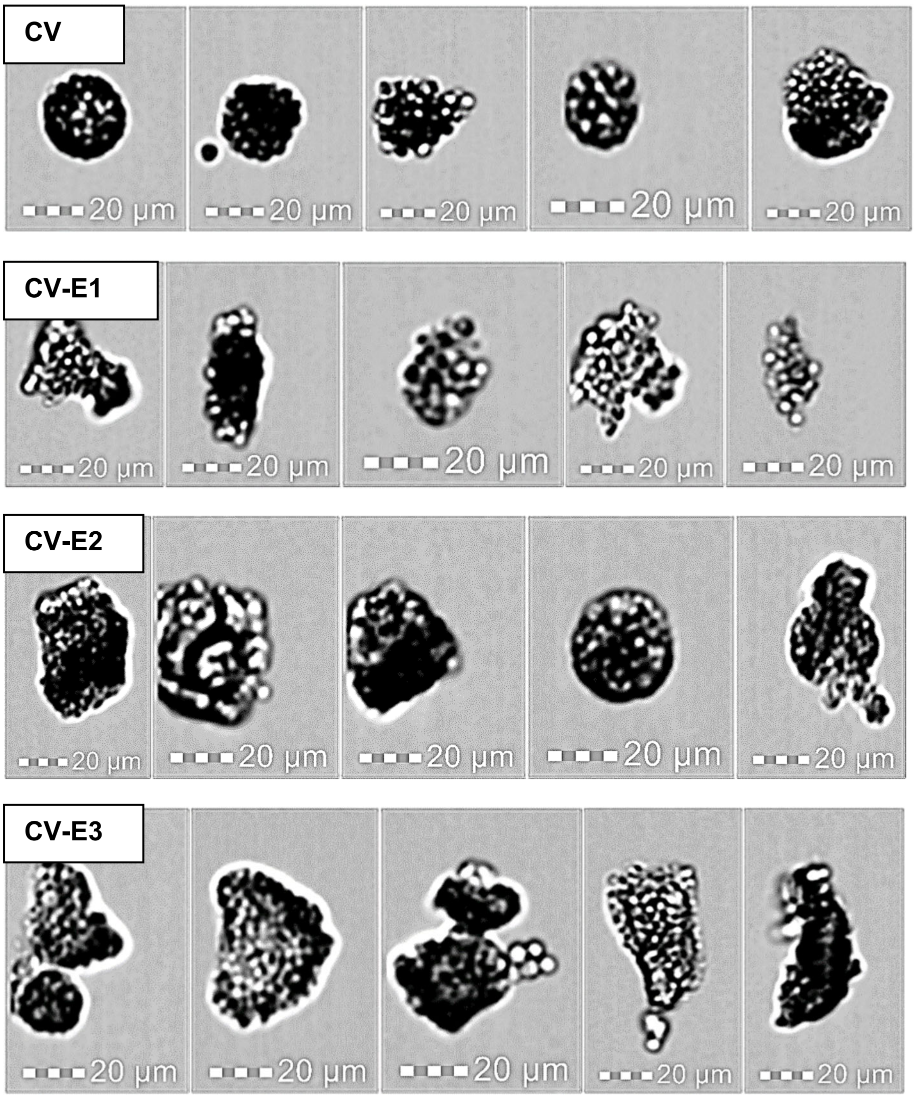


**Figure S6.** Images of *Chlorella vulgaris* powder (CV), *Chlorella vulgaris* powder after sonication (CV-E1), *Chlorella vulgaris* powder after Soxhlet extraction (CV-E2) and *Chlorella vulgaris* powder after ultrasound-assisted solvent extraction (CV-E3) dispersed in buffer (pH 2.0) obtained using a flow cytometer in a visible light channel (out of 1000 images, some of the population with a particle area of 50-100 µm$^2$ were selected).

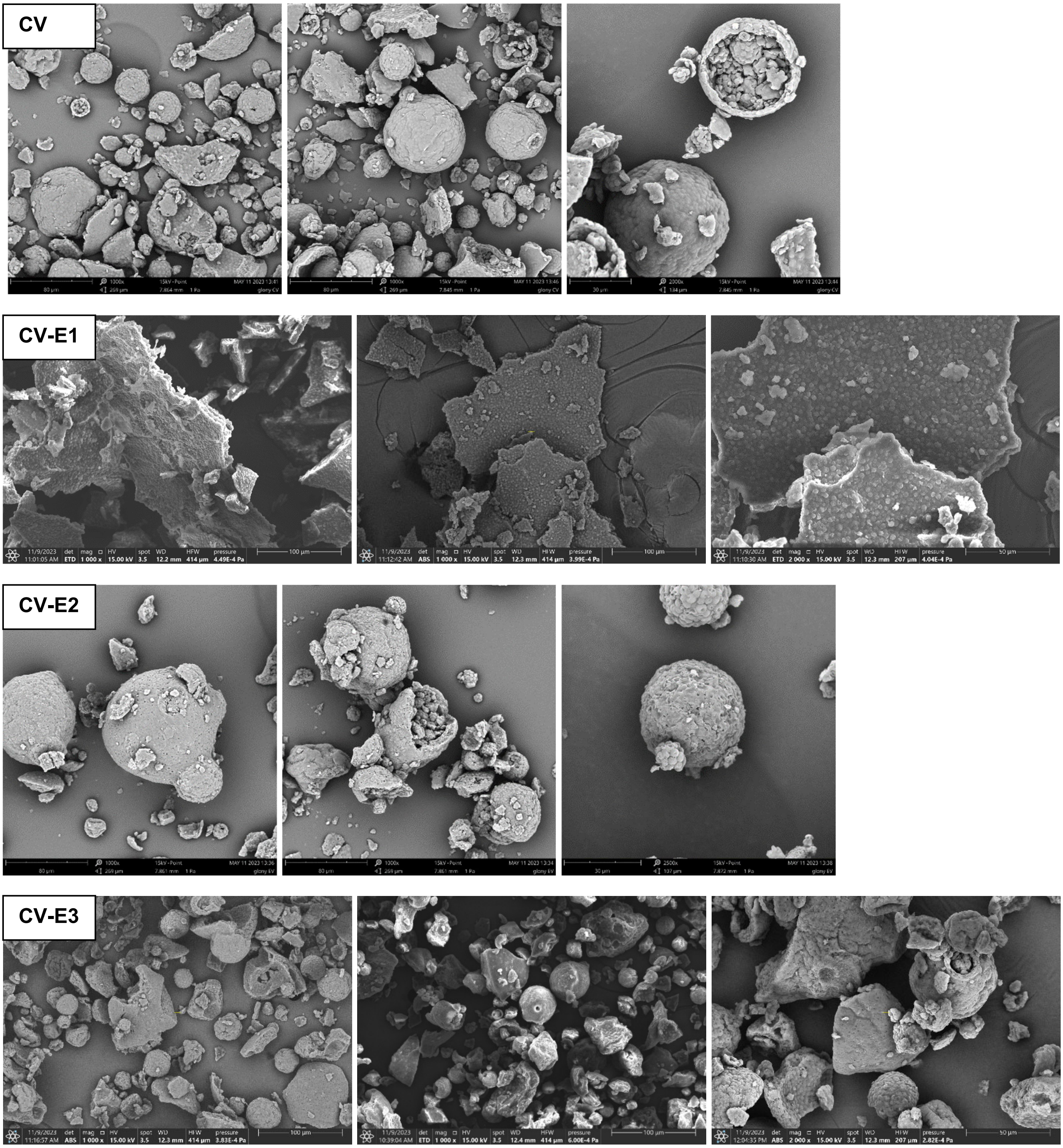


**Figure S7.** SEM images of *Chlorella vulgaris* powder (CV), *Chlorella vulgaris* powder after sonication (CV-E1), *Chlorella vulgaris* powder after Soxhlet extraction (CV-E2) and *Chlorella vulgaris* powder after ultrasound-assisted solvent extraction (CV-E3), magnification 1000x, 2000x (2500x for CV2-2).

**Morphological Characteristics of Sorbents and the Efficiency of Extraction of Lipids and Pigments.**

**Table S6.** Comparison of the morphological characteristics of *Chlorella* powder and derived sorbents using flow cytometry (sorbent solutions in pH 2.0 buffer, 0.5 g/L), gating was applied to exclude outliers and debris. Values are the mean % of total count ± SEM, *– significant difference compared with intact *Chlorella* powder (CV), $p < 0.05$ (Kruskal-Wallis, post-hoc Dunn's test).

| | **CV** | **CV-E1** | **CV-E2** | **CV-E3** |
|---|---|---|---|---|
| **Variables** | **Width** | | | |
| **1-5 µm** | 21.1 ± 1.41 | 36.6 ± 0.53 * | 30.2 ± 2.78 | 21.1 ± 0.74 |
| **5-10 µm** | 50.7 ± 2.28 | 44.0 ± 2.09 | 44.3 ± 1.89 | 39.6 ± 2.07 * |
| **10-50 µm** | 28.2 ± 0.74 | 19.3 ± 1.04 | 25.5 ± 5.58 | 39.3 ± 1.85 |
| | **Height** | | | |
| **1-5 µm** | 10.0 ± 0.19 | 20.8 ± 0.97 * | 18.7 ± 1.76 | 11.6 ± 0.30 |
| **5-10 µm** | 36.3 ± 1.91 | 39.7 ± 1.98 | 35.1 ± 3.13 | 28.6 ± 2.12 |
| **10-50 µm** | 53.6 ± 2.44 | 39.5 ± 1.41 | 46.2 ± 6.85 | 59.9 ± 1.76 |
| | **Roundless** | | | |
| **Round (aspect ratio 0.8-1)** | 28.7 ± 1.60 | 28.7 ± 1.38 | 18.7 ± 1.10 | 18.1 ± 0.12 |
| **Irregular shaped (aspect ratio 0.2-0.8)** | 33.0 ± 1.32 | 36.2 ± 1.44 | 26.0 ± 2.48 | 30.7 ± 0.71 |

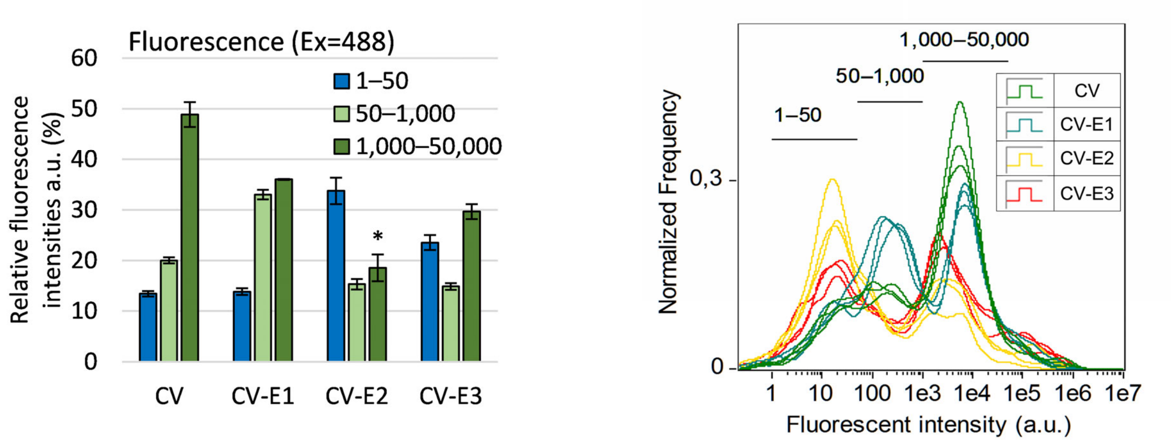


(**a**) (**b**)

**Figure S8.** The comparison of fluorescence intensity of chlorophyll autofluorescence of *Chlorella* powder (CV) and derived sorbents (CV-E1, CV-E2 and CV-E3), Ex=488 nm, 640–745 nm band obtained using flow cytometry (**a**) (gating was applied to exclude outliers and debris, shows on (**b**) part of figure). Populations of particles with different relative fluorescence intensities (1–50, 50–1000 and 1000–50000 a.u.) were detected and determined using the same coordinates for all groups. Values are the mean ± SEM; *- significant difference compared with intact *Chlorella* powder (CV) at $p < 0.05$ (Kruskal-Wallis, post-hoc Dunn's test).

## References


1. de Luna, M.D.G.; Murniati; Budianta, W.; Rivera, K.K.P.; Arazo, R.O. Removal of sodium diclofenac from aqueous solution by adsorbents derived from cocoa pod husks. *J. Environ. Chem. Eng.* **2017**, *5*, 1465–1474.
2. Larous, S.; Meniai, A.-H. Adsorption of Diclofenac from aqueous solution using activated carbon prepared from olive stones. *Int. J. Hydrogen Energy* **2016**, *41*, 10380–10390.
3. Jodeh, S.; Abdelwahab, F.; Jaradat, N.; Warad, I.; Jodeh, W. Adsorption of diclofenac from aqueous solution using Cyclamen persicum tubers based activated carbon (CTAC). *J. Assoc. Arab Univ. Basic Appl. Sci.* **2016**, *20*, 32–38.
4. Baccar, R.; Sarrà, M.; Bouzid, J.; Feki, M.; Blánquez, P. Removal of pharmaceutical compounds by activated carbon prepared from agricultural by-product. *Chem. Eng. J.* **2012**, *211–212*, 310–317.
5. Bagheri, A.; Abu-Danso, E.; Iqbal, J.; Bhatnagar, A. Modified biochar from Moringa seed powder for the removal of diclofenac from aqueous solution. *Environ. Sci. Pollut. Res.* **2020**, *27*, 7318–7327.
6. Hifney, A.F.; Zien-Elabdeen, A.; Adam, M.S.; Gomaa, M. Biosorption of ketoprofen and diclofenac by living cells of the green microalgae Chlorella sp. *Environ. Sci. Pollut. Res.* **2021**, *28*, 69242–69252.
7. Moreno-Castilla, C. Adsorption of organic molecules from aqueous solutions on carbon materials. *Carbon N. Y.* **2004**, *42*, 83–94.
